# Pedestrian Injury and Human Behaviour: Observing Road-Rule Violations at High-Incident Intersections

**DOI:** 10.1371/journal.pone.0021063

**Published:** 2011-06-15

**Authors:** Jonathan Cinnamon, Nadine Schuurman, S. Morad Hameed

**Affiliations:** 1 Department of Geography, Simon Fraser University, Burnaby, British Columbia, Canada; 2 Trauma Services, Vancouver General Hospital, Vancouver, British Columbia, Canada; The University of South Wales, Australia

## Abstract

**Background:**

Human behaviour is an obvious, yet under-studied factor in pedestrian injury. Behavioural interventions that address rule violations by pedestrians and motorists could potentially reduce the frequency of pedestrian injury. In this study, a method was developed to examine road-rule non-compliance by pedestrians and motorists. The purpose of the study was to examine the potential association between violations made by pedestrians and motorists at signalized intersections, and collisions between pedestrians and motor-vehicles. The underlying hypothesis is that high-incident pedestrian intersections are likely to vary with respect to their aetiology, and thus are likely to require individualized interventions – based on the type and rate of pedestrian and motorist violation.

**Methods:**

High-incident pedestrian injury intersections in Vancouver, Canada were identified using geographic information systems. Road-rule violations by pedestrians and motorists were documented at each incident hotspot by a team of observers at several different time periods during the day.

**Results:**

Approximately 9,000 pedestrians and 18,000 vehicles were observed in total. In total for all observed intersections, over 2000 (21%) pedestrians committed one of the observed pedestrian road-crossing violations, while approximately 1000 (5.9%) drivers committed one of the observed motorist violations. Great variability in road-rule violations was observed between intersections, and also within intersections at different observation periods.

**Conclusions:**

Both motorists and pedestrians were frequently observed committing road-rule violations at signalized intersections, suggesting a potential human behavioural contribution to pedestrian injury at the study sites. These results suggest that each intersection may have unique mechanisms that contribute to pedestrian injury, and may require targeted behavioural interventions. The method described in this study provides the basis for understanding the relationship between violations and pedestrian injury risk at urban intersections. Findings could be applied to targeted prevention campaigns designed to reduce the number of pedestrian injuries at signalized intersections.

## Introduction

Road-traffic collisions are responsible for 1.2 million deaths and as many as 50 million injuries annually according to estimates by the World Health Organization [1]. Although death and injury due to road-traffic collisions have decreased in recent years in many high-income countries, their burden remains a large contributor to overall mortality and morbidity. Furthermore, for the world as a whole, road-traffic collisions are projected to be the fourth leading cause of disability adjusted life-years (DALYs) lost by 2030, responsible for 4.2% of total DALYs [2]. In the US in 2009, there were approximately 4,000 deaths and 60,000 injuries due to pedestrian-motor vehicle collisions [3]. Pedestrian injury comprises a substantial proportion of total road-traffic injuries around the world. In fact, there is some evidence to suggest that pedestrian injury is increasing as a proportion of total road-traffic injuries. For instance, in Canada between 2003 and 2007, serious pedestrian injury increased each year as a proportion of total road-traffic injuries, from 9.8% to 12.2% over the 5 year span [4], [5]. Debates as to the significance of this increase aside, enhancing pedestrian safety on roadways should be a prime concern in both the public health and roadway engineering realms. The fact that walking is increasingly promoted for environmental and personal health reasons only fortifies the argument for improving pedestrian safety.

Addressing the risk factors associated with pedestrian injury could help to reduce this persistent public health burden, however, greater understanding of these risk factors is required in order to develop effective pedestrian safety campaigns. Geographic information systems (GIS) are immensely helpful for these purposes, including for a large number of injury prevention tasks, such as; assembling injury and socio-demographic data, analyzing spatial patterns, and visualizing results [6], [7]. In addition to these established applications of this technology, GIS can also be used to inform prevention activities, most notably through identifying specific locations to target prevention efforts. In this study, A GIS approach was used to identify high-incident pedestrian injury locations (hotspots) in Vancouver, Canada. Then, a method was developed to elucidate the potential contribution of violations committed by roadway-users on pedestrian injury at the hotspots, in order to provide evidence that could lead to interventions that target pedestrian or motorist behaviour.

### Human behaviour and pedestrian injury

The pedestrian injury event can result from a single factor, or the complex interplay of multiple contributing factors, both human and environmental. Increasing numbers of studies have highlighted the environmental and demographic connections with pedestrian injury [8], [9], [10]. Human behaviour is another extremely important factor in pedestrian injury [11], however, less attention has been paid to this constituent of pedestrian-motor vehicle collisions. Typically there are actions or behaviours committed by motorists or pedestrians which set off the collision event [12]. Thus, fault in a collision can be attributed to pedestrians or motorists, however, there is conflicting evidence regarding the distribution of fault between the two groups. For the parties involved in a pedestrian-vehicle collision, fault can be assigned by examining violations of roadway legislation. Common violations committed by motorists that could contribute to a pedestrian collision include failure to yield to pedestrians, speeding, and disobeying traffic signs and signals; for pedestrians, failure to yield to vehicles, crossing against a pedestrian signal, or crossing outside of designated markings are frequent violations [13], [14]. In addition to legal violations, non-legal considerations such as negligence or inattention can be used to ascribe fault to either party [14]. Results of a study by Kim *et al.* [12] provides useful information regarding the breakdown of fault between pedestrians and motorists, and detailed information on subgroups of pedestrians and motorists. Fault was determined by violations of legislation (jaywalking) and non-legal considerations (misjudgement and inattention), as recorded in a police crash dataset. Overall, motorists were over 12 times more likely to be at fault in pedestrian-motor vehicle collisions in Hawaii, with male drivers comprising two-thirds of at-fault cases. For the cases in which pedestrians were at-fault, almost 70% were male. Furthermore, male jaywalkers under the influence of alcohol were over 10 times more likely to be at fault than other groups. The authors suggest that even though determining fault may be difficult, “identification of those at-fault can assist in the determination of where to focus efforts of enforcement or educational programs” (p. 2048). On the other hand, a US study found that pedestrians were more likely to be at fault than drivers in both Washington, DC and Baltimore [15]. Police crash data were coded according to precipitating factors leading to the injury event, including legal violations by pedestrians (crossing against the light) and motorists (failure to stop for red lights or stop signs). Inattention and distraction by pedestrians and motorists also played a role in ascribing fault. However, a study in Saudi Arabia found that motorists and pedestrians bore equal responsibility for collisions [16]. Using police records, fault was assigned if a pedestrian (crossing outside of designated markings) or motorist (speeding) violation occurred, or if inattention was a factor for either party. This conflicting evidence may suggest that culpability is highly variable, and may be influenced by the specific attributes of the city or country (culture of safety and enforcement for example), or indeed by the characteristics of the exact injury location (e.g. presence of pedestrian infrastructure, speed limit, local land-use, demographic composition, etc.).

Human behavioural factors such as failure to observe roadway regulations by both pedestrians and motorists clearly contributes to pedestrian-motor vehicle collisions [17]. For motorists, driving behaviour and road-rule compliance have been identified as major contributors to pedestrian injury. Excessive speed, and failure to yield have been cited as common motorist-contributed factors [14]. For example, Preusser *et al.* [15] found that failure by drivers to yield to pedestrians when turning at intersections was a factor in many pedestrian injuries. In Canada from 2002 to 2004, 40% of pedestrian fatalities at intersections were caused by a driver failing to yield the right-of-way or disobeying a traffic sign or signal [18]. Faster vehicle speeds are also implicated in pedestrian-motor vehicle collisions, in part because stopping distance increases in relation to vehicle speed [19]. Faster speed is associated with increased injury severity and fatality; one study found that the risk of pedestrian fatality was 5 times greater at speeds of 50 km/h versus 30 km/h [20]. It is possible that excessive speed and signal non-compliance at intersections may be attributed to the existence of a ‘dilemma zone’ – the area at the approach to an intersection in which a driver has to choose between increasing speed or braking suddenly in order to comply with traffic signal regulations [11]. Driver distraction is also a factor in motor-vehicle collisions. Distractions include technologies such as mobile phones, GPS navigation systems, and audio systems, and seemingly innocuous actions such as eating, smoking, and conversing with passengers [21]. Harbluk *et al.* [22] examined the change in drivers' cognitive abilities as tasks of varying complexity were communicated to them via a hands-free mobile phone. The drivers' visual scanning movements were recorded; as the complexity of the task increased, drivers made significantly fewer eye movements, looked less at the sides of the street for hazards such as pedestrians, and spent less time inspecting instruments and their rear-view mirror. For pedestrians, unsafe road crossing behaviour and non-compliance with road-rules is also a major contributor to pedestrian injury. In some cases, pedestrians are struck by vehicles as a result of knowingly disobeying road crossing rules, however, there is also evidence to suggest that pedestrians may not have full knowledge of right-of-way rules and other road crossing responsibilities [17]. Failure to yield right-of-way, and alcohol impairment are common pedestrian-contributed factors [14]. Modern distractions such as mobile phones and personal music players may also be responsible for pedestrians not complying with road rules [23]. A study set on a university campus [24] examined the effects of talking on a mobile phone on pedestrian awareness and road crossing safety. Results found that those talking on the phone exhibited lower awareness of their surroundings, and crossed unsafely into traffic significantly more than pedestrians not using a phone. In addition, adverse weather conditions may play a role – a recent study has suggested that pedestrians are more to likely become impatient and engage in risky crossing behaviours as outdoor temperatures decrease [25].

### Pedestrian injury prevention

Injury prevention countermeasures aimed at pedestrian safety are described as either active or passive. Active countermeasures include – for both drivers and pedestrians – *education* regarding the safe use of the road area and *enforcement* of road-rules, while passive countermeasures centre on *engineering* solutions, including, modification of the roadway and implementation of traffic-calming solutions in the interest of pedestrian safety [26], [27]. These three E's of traffic safety – education, enforcement, and engineering – are the primary tools available for pedestrian injury prevention.

Pedestrian injury prevention programs vary greatly, from nationwide programs focused on educating pedestrians about personal safety (an active intervention), to upgrades of local injury hotspots designed to redress an engineering defect that was deemed responsible for a high number of injuries at a specific location (a passive intervention). The scale and type of intervention employed for a prevention program depends on the underlying cause of the problem, in addition to other factors including, the availability of funds for prevention, and political will [28].

Generalized, large scale injury interventions are likely to be useful prevention tools, however, they may be less effective in some situations, such as for addressing a high-risk injury location or high-risk group [29]. In many cases, targeted, focused interventions are required to address these pressing issues. This notion has likely contributed to the increased focus in recent years on community-based injury prevention. It has been observed that the effectiveness of community-based injury prevention programs vary temporally and spatially [26]. In other words, what has worked in one location at a specific point in time will not necessarily prove effective in another location or at another point in time. What is required, then, are mechanisms that allow for identifying appropriate prevention alternatives for a certain location at a specific time. For pedestrian injury interventions specifically, Heinonen and Eck [30] suggest that responses must be tailored to local circumstances and should be based on reliable analysis of local conditions.

Modifying pedestrian and motorist roadway behaviours is a common goal of large generalized prevention programs (e.g. through education campaigns), however, behavioural interventions aimed at specific groups or locations are less common, perhaps because of the perceived difficulty of obtaining evidence regarding local roadway users' behaviours. This paper addresses the need for evidence of locally-specific factors that contribute to pedestrian injury. In response to the uncertain effectiveness of generalized pedestrian injury prevention [e.g. 31], a method was developed that could be used to inform the development of interventions that target road rule violations at pedestrian injury hotspot intersections. The method was designed with the goal of providing an easy to implement strategy for community-based injury prevention groups wishing to understand and address a localized pedestrian injury problem. A main hypothesis for this study is that the behavioural contributors to pedestrian injury are likely to vary between hotspots, suggesting the need for individualized responses.

## Methods

In this study, a method was developed to observe violations of road rules by pedestrians and motorists at high incident pedestrian injury locations. The study demonstrates a simple method that could be applied by community injury prevention groups to understand the potential role of pedestrians and motorist violations, which could be useful for designing prevention programs in the local area. First, intersection-level pedestrian injury hotspots in the City of Vancouver were identified using GIS. Next, in-person team surveys of hotspots were conducted to examine violations by motorists and pedestrians and total volume of vehicle and pedestrian traffic, in an effort to elucidate the underlying behavioural mechanisms of pedestrian injury at each hotspot. Signalized intersections were the focus of this study because they have been identified as one of the most common sites of pedestrian-vehicle collisions within the road network [32], and they were the setting for a majority of the incidents recorded in the dataset examined for this study.

Six years (2000 to 2005 inclusive) of pedestrian injury data were extracted from the Insurance Corporation of British Columbia's (ICBC) pedestrian injury dataset. The ICBC dataset consists of information regarding all incidents reported to the provincial automobile insurance corporation. To determine pedestrian injury hotspots, these data were mapped using ArcGIS 9.2 [33]. Incidents that occurred at midblock locations were removed, as the study was restricted to incidents occurring at intersection locations. A smoothed map was produced to facilitate visualization of high incident intersections using a kernel density smoothing function. A search distance of 100 metres was chosen as it best represented individual incident locations. These methods were first demonstrated in an earlier study that focused on the contribution of the built-environment to pedestrian injury at hotspots in Vancouver [8]. For the purposes of this study, the eight intersection hotspots with the highest number of incidents over the study period were considered for analysis (number of incidents in parentheses); Hastings and Main (16), Broadway and Fraser (11), Georgia and Burrard (11), Hastings and Commercial (11) Hastings and Carrall (11), Broadway and Commercial (10), Hastings and Gore (10), and Howe and Davie (9). The set of eight intersections had similar grid layouts, standard two-directional pedestrian crosswalks (i.e. no pedestrian scramble crossings), and each was located on a ‘major arterial’ route. Five of eight locations had 12 approach lanes, two had 9 (Hastings and Carrall, Hastings and Gore), and one had 8 (Howe and Davie). One of the eight intersections (Howe and Davie) was situated on a one-way street (Howe) in one direction; the remainder were two-way streets in all directions. All eight intersections had fixed traffic signal cycles (i.e. each phase completes the full cycle at all times), with green, yellow, and red light phasing for vehicles, and walk, flashing hand, and steady hand phasing for pedestrians. The British Columbia Motor Vehicle Act (MVA) [34] outlines the regulations for traffic and pedestrian signalization at intersections in the province. Pedestrians must adhere to the pedestrian signal phasing regulations as set out in the MVA; *walk* (pedestrian has right of way to cross road within designated crosswalk area), *flashing hand* (pedestrian must not enter roadway or must complete crossing if already started), and *steady hand* (pedestrian must not enter roadway). Motorists must follow the regulations for traffic signal phasing; *green light* (motorist may enter the intersection), *yellow light* (motorist must stop before entering the intersection unless it is unsafe to do so), and *red light* (motorists must not enter the intersection).

Next, violations of the MVA that pertain to intersection signalization were observed (henceforth referred to simply as violations). Teams of five people surveyed eight intersections at three different time periods, morning rush-hour (07:00–09:00), off-peak (10:00–12:00), and evening rush-hour (16:00–18:00). Each site could be visited for any 20-minute window during each of the three time periods. Intersection observations took place on midweek days in November 2009. All intersections were observed on the same day for each time period (e.g. all morning rush hour observations were recorded on the same day for all intersections). Two people were responsible for counting pedestrian violations at intersections. The three violations recorded were; entering the roadway to cross the intersection during the flashing hand phase, entering the roadway during the steady hand phase, and crossing outside of a designated crossing area. One person recorded the motorist violations. Two violations were recorded; entering the intersection during the yellow light phase (note, this is not a violation of the MVA if it is unsafe safe for the driver to stop), and entering during the red light phase. In addition to observing violations, two persons counted the total volume of pedestrians and one person counted the total number of motorists in order to contextualize the number of violations, which will allow for comparisons to other sites and with future observations. All persons involved in the intersection observation portion of the study received task-specific training materials and verbal instructions. A pilot test of the methods was conducted prior to the data collection to ensure the data could be collected by a team of five people with the abovementioned tasks. The methods used to observe pedestrians are similar to a study by King *et al.* [35], which examined the risk associated with illegal road crossing by pedestrians, and a study of motorist and pedestrian behaviours at pedestrian crosswalks by Kim *et al.* [36].

For all observations, a small degree of latitude was afforded to both pedestrians and drivers for pragmatic reasons. For pedestrian violations, a flexibility of two seconds was allowed for pedestrians entering the roadway to cross the intersection after the walk phase had ended. No affordance was allowed for pedestrians entering the roadway on the steady-hand phase. Persons crossing more than 20 metres from the crossing area markings were not included, as they were considered to be crossing mid-block. Also, when counting pedestrians crossing within designated markings, one metre of leeway was allowed on either side of the designated crossing areas, and leaving the crossing area momentarily when entering or exiting the roadway was not considered a violation of road rules. Motorists entering the intersection two seconds or less after the initiation of the yellow light phase were not considered to be in violation of the MVA, because in some cases it may not be safe to stop suddenly at the immediate onset of the yellow phase. For the purposes of the data collection, motorists entering more than two seconds after the yellow light phase started were considered to be committing a violation. No affordance was allowed for motorists entering the intersection on a red light.

## Results

**Figure 1:**
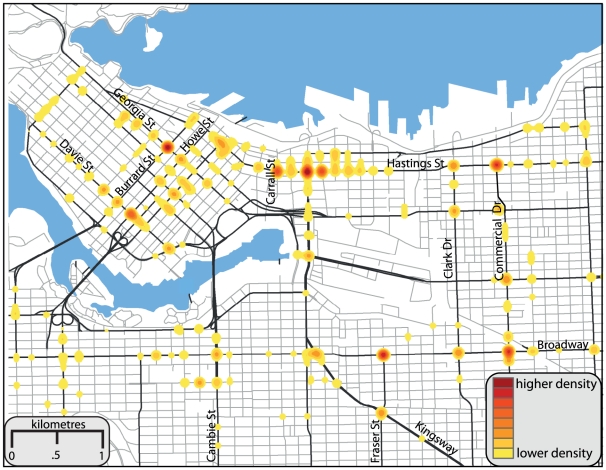
Pedestrian injury hotspot intersections in the City of Vancouver. The top eight pedestrian injury intersection hotspots were (number of incidents in parentheses): Hastings and Main (16), Broadway and Fraser (11), Georgia and Burrard (11), Hastings and Commercial (11) Hastings and Carrall (11), Broadway and Commercial (10), Hastings and Gore (10), and Howe and Davie (9).

**Figure 2:**
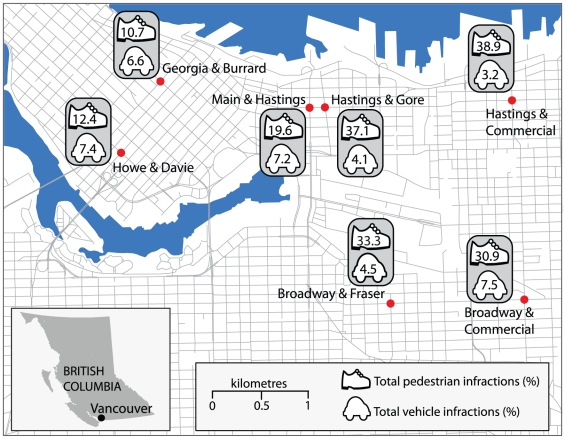
Total pedestrian and motorist violations observed at intersection hotspots. Pedestrian and motorist volume and road rule violations were recorded at the top seven high-incident intersections. This map highlights the total combined violations as a proportion of total volume, for pedestrians and motorists. Great variation was observed between hotspots, for example, at Hastings and Commercial almost 40% of pedestrians committed one of the observed road-crossing violations, while only 12% did at Howe and Davie. Meanwhile, just 3.2% of motorists committed a violation at Hastings and Commercial, but 7.4% of motorists did at Howe and Davie.

Figure 1 illustrates the pedestrian injury hotspot intersections in the City of Vancouver for the 6 year study period. Eight Vancouver intersections were surveyed at three different times of day. However, one intersection – East Hastings St. and Carrall St. – was excluded from analysis as it was blocked in one direction for maintenance during the observation periods and could thus not be compared to the seven other normally functioning intersections. Figure 2 highlights the location and total pedestrian and motorist violations observed during this study at the seven intersections included in the analysis. Table 1 breaks down the results for all pedestrian and motorist violations observed, for all observation periods separately and for the total observations combined. Overall, 9,808 pedestrians and 17,874 vehicles were observed. For all pedestrians observed at all intersections, 8% crossed outside of the designated crossing area; this ranged from just 0.8% at Howe St. and Davie St. to 24.7% at East Hastings St. and Commercial Dr. Overall, 9.8% of pedestrians observed entered the crosswalk area during the flashing hand phase, this varied from 7.2% at Georgia St. and Burrard St. to 15.8% at Broadway and Commercial Dr. Just 3.2% of all observed pedestrians entered on the steady hand; this ranged from 0.5% at Georgia and Burrard, to 9.7% at East Hastings St. and Gore St. Overall, 2,069 (21%) pedestrians committed one of the observed road-crossing violations, ranging from just over 12% at Howe St. and Davie St., to 39% at the East Hastings St. and Commercial Dr. intersection. For motorists, 4.6% overall entered an intersection during the yellow signal phase; this varied between intersections, from 2.5% at East Hastings St. and Commercial Dr., to 6.6% at Howe St. and Davie St. Just 1.3% of motorists entered an intersection during the red signal phase for all intersections, this varied from 0.7% at East Hastings St. and Commercial Dr., to 2% at Broadway and Commercial Dr. Overall, 1,051 (5.9%) of motorists committed one of the observed violations, ranging from 3.2% at East Hastings St. and Commercial Dr., to 7.5% at Broadway and Commercial Dr.

**Table 1:** Violations and volumes of pedestrians and motorists at pedestrian injury hotspots.

		Pedestrians					Motor-vehicles			
Location	Observation time	Total ped. volume	Cross outside markings	Enter on flashing hand	Enter on steady hand	Total ped. violations	Total vehicle volume	Enter on yellow light	Enter on red light	Total vehicle violations
Hastings & Main 1	AM peak	292	30 (10.3)	24 (8.2)	15 (5.1)	69 (23.6)	650	39 (6.0)	10 (1.5)	49 (7.5)
Hastings & Main 2	AM off-peak	742	23 (3.1)	93 (12.5)	24 (3.2)	140 (18.9)	612	41 (6.7)	12 (2.0)	53 (8.7)
Hastings & Main 3	PM peak	637	28 (4.4)	57 (8.9)	34 (5.3)	119 (18.7)	762	35 (4.6)	9 (1.2)	44 (5.8)
**Hastings & Main Total**	**Combined**	**1,671**	**81 (4.9)**	**174 (10.4)**	**73 (4.4)**	**328 (19.6)**	**2,024**	**115 (5.7)**	**31 (1.5)**	**146 (7.2)**
Broadway & Commercial 1	AM peak	575	142 (24.7)	90 (15.7)	15 (2.6)	247 (43.0)	1,260	73 (5.8)	25 (2.0)	98 (7.8)
Broadway & Commercial 2	AM off-peak	619	125 (20.2)	59 (9.5)	7 (1.1)	191 (30.9)	1,028	48 (4.7)	19 (1.8)	67 (6.5)
Broadway & Commercial 3	PM peak	1,104	55 (5.0)	213 (19.3)	4 (0.4)	272 (24.6)	1,405	83 (5.9)	30 (2.1)	113 (8.0)
**Broadway & Commercial Total**	**Combined**	**2,298**	**322 (14)**	**362 (15.8)**	**26 (1.1)**	**710 (30.9)**	**3,693**	**204 (5.5)**	**74 (2)**	**278 (7.5)**
Broadway & Fraser 1	AM peak	60	9 (15.0)	7 (11.7)	4 (6.7)	20 (33.3)	785	24 (3.1)	6 (0.8)	30 (3.8)
Broadway & Fraser 2	AM off-peak	100	13 (13.0)	19 (19.0)	7 (7.0)	39 (39.0)	743	9 (1.2)	5 (0.7)	14 (1.9)
Broadway & Fraser 3	PM peak	80	9 (11.3)	5 (6.3)	7 (8.8)	21 (26.3)	857	42 (4.9)	21 (2.5)	63 (7.4)
**Broadway & Fraser Total**	**Combined**	**240**	**31 (12.9)**	**31 (12.9)**	**18 (7.5)**	**80 (33.3)**	**2,385**	**75 (3.1)**	**32 (1.3)**	**107 (4.5)**
Georgia & Burrard 1	AM peak	1,205	32 (2.7)	90 (7.5)	6 (0.5)	128 (10.6)	1,230	62 (5.0)	20 (1.6)	82 (6.7)
Georgia & Burrard 2	AM off-peak	902	26 (2.9)	56 (6.2)	3 (0.3)	85 (9.4)	1,047	48 (4.6)	19 (1.8)	67 (6.4)
Georgia & Burrard 3	PM peak	1,401	49 (3.5)	105 (7.5)	9 (0.6)	163 (11.6)	1,241	64 (5.2)	19 (1.5)	83 (6.7)
**Georgia & Burrard Total**	**Combined**	**3,508**	**107 (3.1)**	**251 (7.2)**	**18 (0.5)**	**376 (10.7)**	**3,518**	**174 (5)**	**58 (1.6)**	**232 (6.6)**
Hastings & Commercial 1	AM peak	80	24 (30.0)	7 (8.8)	3 (3.8)	34 (42.5)	1,028	26 (2.5)	0 (0.0)	26 (2.5)
Hastings & Commercial 2	AM off-peak	70	21 (30.0)	12 (17.1)	8 (11.4)	41 (58.6)	718	3 (0.4)	2 (0.3)	5 (0.7)
Hastings & Commercial 3	PM peak	125	23 (18.4)	6 (4.8)	3 (2.4)	32 (25.6)	1,158	45 (3.9)	18 (1.6)	63 (5.4)
**Hastings & Commercial Total**	**Combined**	**275**	**68 (24.7)**	**25 (9.1)**	**14 (5.1)**	**107 (38.9)**	**2,904**	**74 (2.5)**	**20 (0.7)**	**94 (3.2)**
Hastings & Gore 1	AM peak	112	23 (20.5)	14 (12.5)	25 (22.3)	62 (55.4)	519	12 (2.3)	0 (0.0)	12 (2.3)
Hastings & Gore 2	AM off-peak	506	115 (22.7)	41 (8.1)	39 (7.7)	195 (38.5)	457	13 (2.8)	5 (1.1)	18 (3.9)
Hastings & Gore 3	PM peak	361	55 (15.2)	21 (5.8)	31 (8.6)	107 (29.6)	670	31 (4.6)	7 (1.0)	38 (5.7)
**Hastings & Gore Total**	**Combined**	**979**	**193 (19.7)**	**76 (7.8)**	**95 (9.7)**	**364 (37.1)**	**1,646**	**56 (3.4)**	**12 (0.7)**	**68 (4.1)**
Howe & Davie 1	AM peak	244	1 (0.4)	11 (4.5)	5 (2.0)	17 (7.0)	553	40 (7.2)	4 (0.7)	44 (8.0)
Howe & Davie 2	AM off-peak	273	5 (1.8)	28 (10.3)	17 (6.2)	50 (18.3)	504	29 (5.8)	1 (0.2)	30 (6.0)
Howe & Davie 3	PM peak	320	1 (0.3)	29 (9.1)	7 (2.2)	37 (11.6)	647	43 (6.6)	9 (1.4)	52 (8.0)
**Howe & Davie Total**	**Combined**	**837**	**7 (0.8)**	**68 (8.1)**	**29 (3.5)**	**104 (12.4)**	**1,704**	**112 (6.6)**	**14 (0.8)**	**126 (7.4)**
**Total (all observations)**	**Combined**	**9,808**	**809 (8)**	**987 (9.8)**	**273 (3.2)**	**2,069 (21.1)**	**17,874**	**810 (4.6)**	**241 (1.3)**	**1,051 (5.9)**

**Note: Parentheses denote violations as per cent of total volume.**

In addition to the overall findings at each location, several individual findings are notable. For instance, at Hastings and Commercial, fully 39% of total observed pedestrian crossings resulted in a violation; however, even more striking are the results of the individual observations at this location. During the off peak observation, 30% crossed outside the markings, 17.1% crossed on the flashing hand phase, and 11.4% crossed on the steady hand phase; thus, almost 59% of crossings by pedestrians included one of the observed violations. At Hastings and Gore, more pedestrians crossed on the steady hand phase than the flashing hand phase during the AM peak observation (22.3% crossed on the steady hand phase compared to 12.5% on the flashing hand phase), during the PM peak observation (8.6% steady hand, 5.8% flashing hand), and results were almost equal for both phases during the off peak observation (7.7% steady hand, 8.1% flashing hand).

**Figure 3:**
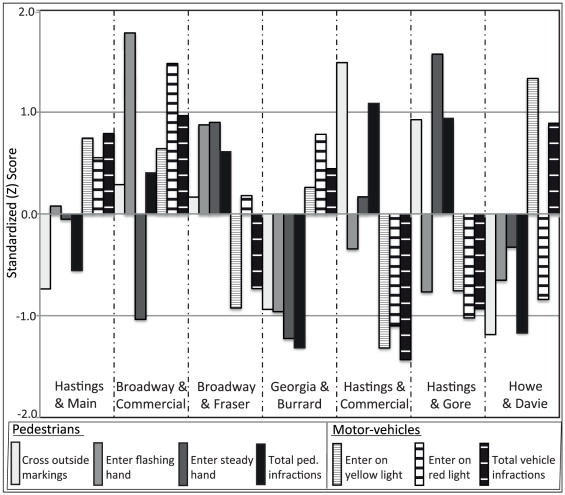
Standardized scores for all violations at seven intersections. Values above 0 are higher than the mean, below 0 are less than the mean. Using this graph, it is possible to visualize the potential contribution of pedestrian and motorist roadway violations. For example, at Georgia and Burrard, all pedestrian violations are less than the overall mean, while all motorist violations are higher than the mean. This may suggest that interventions at this site might be most effective if motorists are targeted.

In Figure 3, the proportional results of each surveyed violation combined for all observation times are standardized using a Z score transformation. This graph allows for the variables to be compared between intersections, based on the overall mean of each violation. Values higher that 0 are above, and values below 0 are less than the overall mean for that violation. All vehicle violations are above the mean at Hastings and Main, Broadway and Commercial, and Georgia and Burrard, and below the mean at Hastings and Commercial and Hastings and Gore. All pedestrian violations are above the mean at Broadway and Fraser, and below the mean at Georgia and Burrard and Howe and Davie.

## Discussion

In this study, GIS methods were used to identify pedestrian injury hotspots. A GIS approach allows researchers to identify the spatial distribution of injury, and then to examine the contextual factors associated with pedestrian injury; specifically, how location interacts with human, social, and environmental factors to influence injury risk. For decision-makers, maps and cartographic visualizations created in a GIS environment are powerful knowledge translation tools for understanding a problem and forcing action on important societal issues. The power of GIS for examining pedestrian injury has increasingly been demonstrated in recent years [37], [38], [39]. Following the identification of hotspots at signalized intersections, violations of road rules by pedestrians and motorists were observed in order to elucidate their potential contribution to pedestrian injury in Vancouver, Canada. Based on the premise that certain high-incident intersections should be targeted individually with regard to safety countermeasures, a simple observational-based method was developed that could be used to determine what types of behaviour-modifying interventions may be most appropriate. The results of this study highlight great variability in violations observed between locations, which may suggest accordance with the hypothesis that hotspots are likely to be dissimilar with respect to their aetiology. In Figure 3, a clear pattern emerges for some of the hotspots in particular. For example, at Georgia and Burrard Sts. the relative values are low for the pedestrian violations and high for the motorist violations. This may indicate that motorists entering the intersection after the green light phase is contributing to pedestrian injury. Interventions that target violations by motorists may be most appropriate in this location. On the other hand – at Hastings and Commercial – pedestrians may be the more likely contributor, as this location boasts the highest proportion of pedestrians committing a violation (almost 40%) coupled with the lowest proportion of motorists committing a violation (3.2%). As such, countermeasures that target violations by pedestrians may be most appropriate at this location. In other cases, it is less clear which group may be responsible; this may suggest that both motorists and pedestrians should be targeted.

Inconsistencies in violations observed between different locations in a city might indicate *actual* variability in the people frequenting each intersection, or it might point to the notion that road users may be *influenced* to commit violations due to characteristics of the surrounding area. For instance, certain types of land use might influence a pedestrian to cross against the signal. The presence of a pedestrian generator such as a public transit hub might influence a pedestrian to commit a violation in order to avoid missing a transit connection. Also, research linking commercial and residential areas, schools, and alcohol establishments to pedestrian injury [8], [9], [10] might suggest these types of land use influence a road user to commit a violation. The seven locations observed in this study diverge with respect to characteristics of the surrounding area. Looking at the local characteristics of some of the more concerning findings from this study, it may be possible to posit alternative explanations. The land use at Hastings and Commercial is predominantly light industrial – which is not typically a significant pedestrian attractor, however several public buses stop at three of the four corners of this intersection. Pedestrians alighting from one bus and then rushing across the street to board another might provide an alternative explanation for the very high proportion of pedestrian violation crossings observed at this location. Hastings and Gore is situated in an area comprised of commercial and residential land uses, and multiple alcohol serving establishments. It is possible that these land uses are influencing pedestrians' to commit a violation. For organizations wishing to determine why violations are occurring at certain locations, a logical next step might be to attempt to understand the variability between locations and assess how local characteristics such as land use contribute to violations. Combining the methods described in this study with an on-site inspection of local characteristics and a survey of road users' attitudes may help to shed further light on the specific contextual factors that influence violation of road rules by pedestrians and motorists.

The present study was not designed to identify local characteristics, however, it may be useful for identifying another potential contributor to pedestrian-vehicle collisions, the total volume of pedestrians and vehicles at certain locations. For example, if little non-compliance is noticed at hotspots, it is possible that hotspots may be associated simply with pedestrian or vehicle volumes based on the notion that greater volume of all road users will provide more opportunities for conflicts between vehicles and pedestrians [40]. Research has confirmed this idea [41], [42], however studies have also documented a tapering off of this relationship at the higher end. In a study of intersections in Florida, Lee and Abdel-Aty [43] found that the likelihood of pedestrian-vehicle conflict is higher at intersections with greater traffic volume. However, the relationship attenuates as traffic volumes increase, suggesting that congestion (very high vehicle volume) reduces the likelihood of conflict. Evidence has also suggested pedestrian injury may be associated with higher volumes of pedestrians [39], [44]. However, there is also evidence to suggest this relationship diminishes as pedestrian volumes increase [45]. This may have to do with the so-called ‘safety in numbers’ effect, whereby motorists may be influenced to drive slower and with more caution in the presence of elevated pedestrian volumes [46]. In a study in Oakland, California, Geyer *et al.* [47] concluded that the risk of injury for pedestrians decreases as pedestrian volumes increase, and, increases as vehicle volumes increase. Some of the wisdom regarding volumes coming from previous research is echoed in the current study. For example, very low relative pedestrian volumes and high relative vehicle volumes at Hastings and Commercial and Broadway and Fraser could suggest an exposure-related aetiology at these locations, following the findings from the Oakland study. This varying evidence of the effect of volumes on pedestrian injury suggests that the relationship may be non-linear, context-specific, and confounded by other variables. What is needed in particular is more research to understand the combined, interconnected effects of pedestrian and vehicle volumes at hotspots. By focussing on both pedestrian and vehicle volumes, the present study could provide a starting point for examining how different volumes of all road users coalesce to either increase or decrease pedestrian injury risk. For hotspots that appear to be affected by high vehicle or pedestrian volumes, suitable interventions may include engineering solutions to segregate vehicles and pedestrians, or simply, a reduction in the speed limit at these hotspots.

### Modifying roadway-users' behaviours

Despite the fact that choices made by motorists and pedestrians while in the roadway have an obvious effect on pedestrian injury, behavioural-focused injury interventions are rare compared to engineering solutions [48]. Because engineered solutions such as traffic calming are not always feasible or effective, behavioural interventions could be targeted directly at those road users who are committing violations in order to see results [49]. Interventions aimed at changing behaviours focus on reducing the risk of injury through promotion of safe behaviour while operating a vehicle or walking in the roadway [48]. For problem intersections, behavioural interventions can be designed that target pedestrians, motorists, or both groups to emphasise safe behaviours and knowledge of road regulations. With regard to pedestrians, an obvious target for intervention is choices made at the side of the road [50], including where and when to cross an intersection. Harré & Wrapson [51] examined the effects of installation of visual media and provision of rewards for road-rule compliance on pedestrians road-crossing behaviour at five intersections in Auckland. These interventions were successful in reducing the proportion of pedestrians crossing during the red light phase by half. For motorists, proceeding through an intersection after the green light phase is a decision that increases the risk of colliding with a pedestrian. Speeding, carelessness, and distractions are all factors that influence drivers' choices made at intersections, and are clear targets for behavioural intervention. Nasar [52] examined the benefit of an intervention designed to encourage drivers to yield to pedestrians at crosswalks. Signs were held up by volunteers to thank the driver for stopping when compliance was observed, or, to ask the driver to stop next time in the case of non-compliance. This simple intervention was successful in increasing the proportion of drivers stopping for pedestrians at the study crossing, and was also associated with an increase at a nearby crossing that was not subject to the treatment.

These successful examples of intersection-level behavioural interventions underscore the notion that modifying the behaviour of roadway users with respect to safety at trouble spots could reduce the burden of injury. Winston and Jacobsohn's [48] step-by-step behavioural intervention tutorial could be a useful framework for implementing the required interventions. Results could allow for evidence-based decision making by communities that wish to reduce their burden of pedestrian injury. Following an intervention, the method could be applied again in order to understand the potential effects of the program.

Previous research has focused on examining violations using observational techniques. For example, Cambon de Lavalette et al. [53] examined the interplay between environmental factors and the decision-making processes of pedestrians. The aim of this study was to examine how the surrounding environment potentially mediates safe road crossing behaviours. One of the findings of this observational study suggested that violations increase with the absence of crossing signals. King *et al.* [35] attempted to determine the risk of injury for pedestrians that violate crossing signals. The results of the study provided both evidence for the risk of crossing against the signal (approximately 8 times greater than crossing legally), and a method to undertake this type of study. Research has also focused on violations by motorists. For instance, in a study by Yang *et al.* [54], violations at signalized intersections were examined using red-light photo enforcement camera data. Findings from this research suggested that younger drivers were more likely to disobey red lights than other age groups, and red light violations were lower during off-peak times of the day. Kim *et al.* [36] directly observed both pedestrian and motorists at pedestrian crosswalks after the implementation of new pedestrian right of way legislation. Similar to the results of the present study, this study found great variability between locations. Accordingly, the authors state (p. 902): “it may be necessary to develop localized enforcement, education, and engineering solutions. A one size fits all approach, evidently, will not be as effective as a more customized approach to addressing particular locations or at least types of locations.” The present study used simple observational techniques to examine violations by motorists and pedestrians at signalized intersections. A relatively easy to implement strategy was developed and demonstrated that could allow pedestrian injury stakeholders to identify the specific types of behavioural interventions that may be most appropriate for targeting pedestrians or motorists in their community. A community-based pedestrian injury advocacy group in Vancouver, Canada has adapted and utilized the methods described in this study to examine violations on local neighbourhood streets [55]. Using information gleaned from the observations, the group developed overall and site-specific recommendations for improving pedestrian safety in the neighbourhood, including education, engineering, and enforcement solutions.

### Limitations

Several limitations are evident with this study. The method described in this paper is designed to provide greater understanding regarding violations of road rules by pedestrians and motorists, which may be useful for designing targeted interventions. Fault, however, is likely to be speculative. In this case, we enumerated both pedestrian and motorist violations but there may be underlying factors that enhance likelihood of such violations. Findings may be better understood when compared to other research findings; for example, studies of pedestrian unfriendly roadway design at intersections. Another limitation of this study relates to the choice of violations included for analysis and the length of time for hotspot observations. In addition, observing intersections at any 20-minute window within the two-hour time periods may be a limitation if volumes change over this time. These study design choices reflect the restrictions imposed by the funding period and availability of personnel, however, we believe this was adequate in order to demonstrate a methodological framework for providing insight into this lesser known issue. Organizations that wish to undertake a study of human behaviour at intersections may wish to observe for longer periods, add more observation times during the day, or include a different set of violations, non legal considerations, or other aspects of human behaviour deemed appropriate at the specific location.
